# Characterization and Evaluation of Reverse Osmosis Membranes Modified with Ag_2_O Nanoparticles to Improve Performance

**DOI:** 10.1186/s11671-015-1080-3

**Published:** 2015-09-29

**Authors:** Abdullah S. Al-Hobaib, Khalid M. AL-Sheetan, Mohammed Rafi Shaik, Naser M. Al-Andis, M. S. Al-Suhybani

**Affiliations:** Nuclear Science Research Institute, King Abdulaziz City for Science and Technology (KACST), P.O. Box 6086, Riyadh, 11442 Saudi Arabia; Department of Chemistry, College of Science, King Saud University, P.O. Box. 2455, Riyadh, 11451 Kingdom of Saudi Arabia

**Keywords:** Ag_2_O nanoparticles, Interfacial polymerization, RO membrane, Modified membrane, Desalination

## Abstract

The objective of this work was to prepare and characterize a new and highly efficient modified membrane by in situ interfacial polymerization on porous polysulfone supports. The process used *m*-phenylenediamine and trimesoyl chloride in hexane, incorporating silver oxide Ag_2_O nanoparticles of varied concentrations from 0.001 to 0.1 wt%. Ag_2_O nanoparticles were prepared at different sizes varying between 20 and 50 nm. The modified membranes were characterized by X-ray diffraction (XRD), scanning electron microscopy (SEM), energy-dispersive X-ray spectroscopy (EDX), atomic force microscopy (AFM), transmission electron microscopy (TEM), and contact angle measurement. The results showed a smooth membrane surface and average surface roughness from 31 to 74 nm. Moreover, hydrophilicity improved and the contact angle decreased to 41° at 0.009 wt% silver oxide. The performances of the developed membranes were investigated by measuring permeate fluxes and salt rejection capability by passing NaCl solutions (2000 ppm) through the membranes at 225 psi. The results showed that the flux increased from 26 to 40.5 L/m^2^ h, while the salt rejection was high, at 99 %, with 0.003 wt% Ag_2_O nanoparticles.

## Background

Water purification technologies are attracting increasing attention, because of gradually increasing shortages of fresh water and lack of access to safe drinking water. Reverse osmosis (RO) is an important membrane technology for water purification through decontamination of industrial effluents and desalination of sea water. Recently, a number of workers have focused on developing high water flux membranes with low processing cost [1–8]. Several methods have already been developed for RO membrane fabrication including phase inversion [9] and interfacial polymerization (IP) [10]. However, concerted efforts are constantly applied to innovate new efficient supports [11], polymer types [12], and tailor polymer structures [13].

From ancient times, human beings have collected consumable fresh water from rivers, lakes, canals, ponds, and underground aquifers [14]. The sources of fresh water are depleted over time because of several reasons such as growth in population, industrialization, modernization of human societies, and enormous changes in climate. The sources of the fresh water are being contaminated significantly due to agricultural and industrial effluents flowing into lakes, canals, and rivers which are constantly leaking into underground water. These effluents contain many contaminants, such as metals, dyes, pesticides, fertilizers, and biologics, which are harmful to human consumption because of their toxicity [15, 16]. Therefore, human beings are always in search of fresh drinkable water sources for making it available to the consumer. In order to pursue this effort, many physical-chemical methods have been developed and adopted at different times. Some use chemical treatments to remove contaminants from polluted sweet water by adopting various techniques, while others use heat treatment to obtain fresh water through vaporization followed by condensation. These methods are not economically viable, are time consuming, and are environmentally hazardous and unfriendly.

The membrane composition and its subsequent surface chemistry accomplish its interaction with water, thus affecting its wettability. The membrane wettability can be calculated by assessing the contact angle between the membrane surface and a droplet of liquid. Hydrophilic membranes are characterized by the presence of active groups that have the ability to form hydrogen bonds with water and so these membranes have wettability. Generally, higher charge density on a membrane surface is accompanying with higher hydrophilicity of the membrane. Membrane surface morphology also has a considerable effect on flux, fouling as pore size, pore geometry, and pore size distribution particularly at the surface of the membrane [17].

Surface hydrophilicity augmented when the hydrophilic nanoparticles (NPs) were presented onto the surface of the membrane. Enriched water flux came from the hydrophilic membrane surface. Furthermore, water affinitive surface area improved if the nanoparticle had a higher degree of dispersion and also by increasing membrane hydrophilicity and reduce the cross-linking degree of polyamide thin-film composite membrane by competing with *m*-phenylenediamine (MPD), which ensued in a great enhancement of water flux. Further, the decrease of the cross-linking degree could also increase salt passage through the membrane [18, 19].

A great number of RO and nanofiltration (NF) membranes with good decontamination performances and permeate fluxes have been developed [20–23]. RO membranes have very high levels of rejection of inorganic solutes such as monovalent ions, hardness components (e.g., calcium, magnesium), and organic matters such as trihalomethane precursor, pesticides, and deodorants at operating pressures of approximately 0.5–6.0 MPa [24].

An RO membrane is composed of three layers: a bottom layer made of unwoven polyester cloth of thickness 100–200 μm to support the entire membrane, a middle layer consisting of polysulfone (PSF) or polyethersulfone (PES) of thickness 30–50 μm, and a top layer of polyamide (PA) or polyetherimide (PEI), supported by PSF or PES, of average thickness 100–200 nm, which is used to separate solutes from feed water. A PA membrane is prepared on the surface of the middle layer by interfacial polymerization between a diamine moiety of 1,3-phenylenediamine (MPD) in the water phase and an acid chloride moiety of 1,3,5-benzenetricarbonyl trichloride (TMC) in the nonpolar organic phase. An industrial product named FT-30 is one such RO membrane, which has a high level of salt rejection of more than 99 % and fluxes of more than 1 m^3^/(m^2^/day) for 2000 ppm NaCl at 1.55 MPa [25]. Thus, many types of RO membranes have been developed.

Kamada et al. developed polyamide RO membranes with controlled surface morphology by interfacial polymerization of water-soaked MPD with TMC in an organic nonpolar hexane medium on polysulfone ultrafiltration supports [26].

Biofouling and virus penetration are two significant obstacles in water treatment using membrane filtration [27], because both reduce membrane permeability, increase energy costs, and decrease the lifetime of membranes. To effectively remove viruses, nanofiltration or reverse osmosis should be used with low-pressure membranes having anti-biofouling and antiviral properties. The antibacterial properties of silver are well known, and silver nanoparticles (nano-Ag) are now incorporated into polymer membranes, including RO membranes, as well as in a wide variety of consumer products for microbial control [27].

In this study, nano-Ag_2_O was incorporated into RO membranes. Nanosilver oxide incorporation also increases membrane hydrophilicity and reduces the potential for other types of membrane fouling. The objectives of this paper were to prepare and characterize new and highly efficient modified polyamide (PA) membranes incorporating silver oxide (Ag_2_O) nanoparticles. Ag_2_O nanoparticles were prepared at different sizes, varying between 20 and 50 nm.

## Methods

### Materials

The materials and chemicals which were used in this study are of analytical grade as demonstrated in the following: polysulfone supports (PS-20) were purchased from Sepro, USA; *n*-hexane (99 %) was purchased from Oxford Laboratory, India; *m*-phenylenediamine (99 %) (MPD), 1,3,5-benzenetricarbonyl trichloride (TMC), silver nitrate, sodium oleate, triethylene glycol, and *n*-dodecane (99 %) were purchased from Sigma-Aldrich, USA; *n*-cyclohexane, *n*-heptane, and sodium carbonate anhydrous (99 %) were purchased from Scharlau, Spain; ammonium nitrate was purchased from Avonchem Limited, UK; and ultrapure deionized (DI) water was purchased from a Millipore Milli-Q system which was used in all experiments.

### Preparation of Ag_2_O Nanoparticles

Ag_2_O nanoparticles were prepared by the method as follows: Equimolar quantity of silver nitrate 4.61 g (0.0271 mol) was reacted with 8.25 g (0.0271 mol) sodium oleate to yield silver oleate. The silver oleate thus obtained was added to triethylene glycol and heated to reflux temperature while stirring continuously for about 2 h. The reaction mixture was then centrifuged for 10 min and the supernatant was discarded and dried in an oven at 250 °C for 3 h to a powder, which was finely ground to obtain silver oxide nanoparticles [28].

### Preparation of Polyamide Membranes

Polyamide thin-film composite membranes were prepared by immersing a commercial polysulfone product support (PS-20) in an aqueous solution of MPD (2 *v*/*v*%) for 2 min and then the excess MPD solution was removed by pressing the membrane under a rubber roller. The membrane was then immersed in 0.1 % of a TMC and hexane (99 %) solution for 1 min, rinsed with 0.2 % Na_2_CO_3_, washed with DI water, and finally stored in a refrigerator ≈4 °C in DI water prior to use.

### Synthesis of Polyamide/Silver Oxide Nanocomposite Membranes

The silver oxide-polyamide nanocomposite membranes were synthesized similarly to thin-film composite (TFC) membranes, except that silver oxide nanoparticles were added in the 0.001–0.1 *w*/*v*% TMC in *n*-hexane solution beforehand. Various amounts of Ag_2_O nanoparticles were dispersed in TMC-*n*-hexane solution by ultra-sonicating for 60 min at 20 °C. The resultant solution was immediately used for interfacial polymerization (IP) with MPD-soaked PS supports to form the thin-film nanocomposite (TFN) membranes.

### Characterization

#### Scanning Electron Microscopy (SEM)

The morphology and microstructure of the as-synthesized nanocomposite membrane was examined by scanning electron microscope (SEM; FEI Nova-Nano SEM-600, The Netherlands).

#### Atomic Force Microscopy (AFM)

The AFM device was a nanosurf scanning probe-optical microscope (Bruker Corporation). Atomic force microscopy was used to analyze the surface morphology and roughness of the prepared membranes. Small squares of the prepared membranes (approximately 1 cm^2^) were cut and glued on a glass substrate for the analysis.

### Goniometer

Contact angle analysis was performed using a Ramé-Hart Model 250 Standard Goniometer/Tensiometer with drop image advanced software (Ramé-Hart Instrument Co., Succasunna, NJ). A water droplet was placed on a dry, flat homogeneous membrane surface, and the contact angle between the water and membrane was measured until no further change was observed. The average contact angle for distilled water was determined in a series of eight measurements for each of the different membrane surfaces.

### Cross-Flow (Flux and Salt Rejection)

**Fig. 1:**
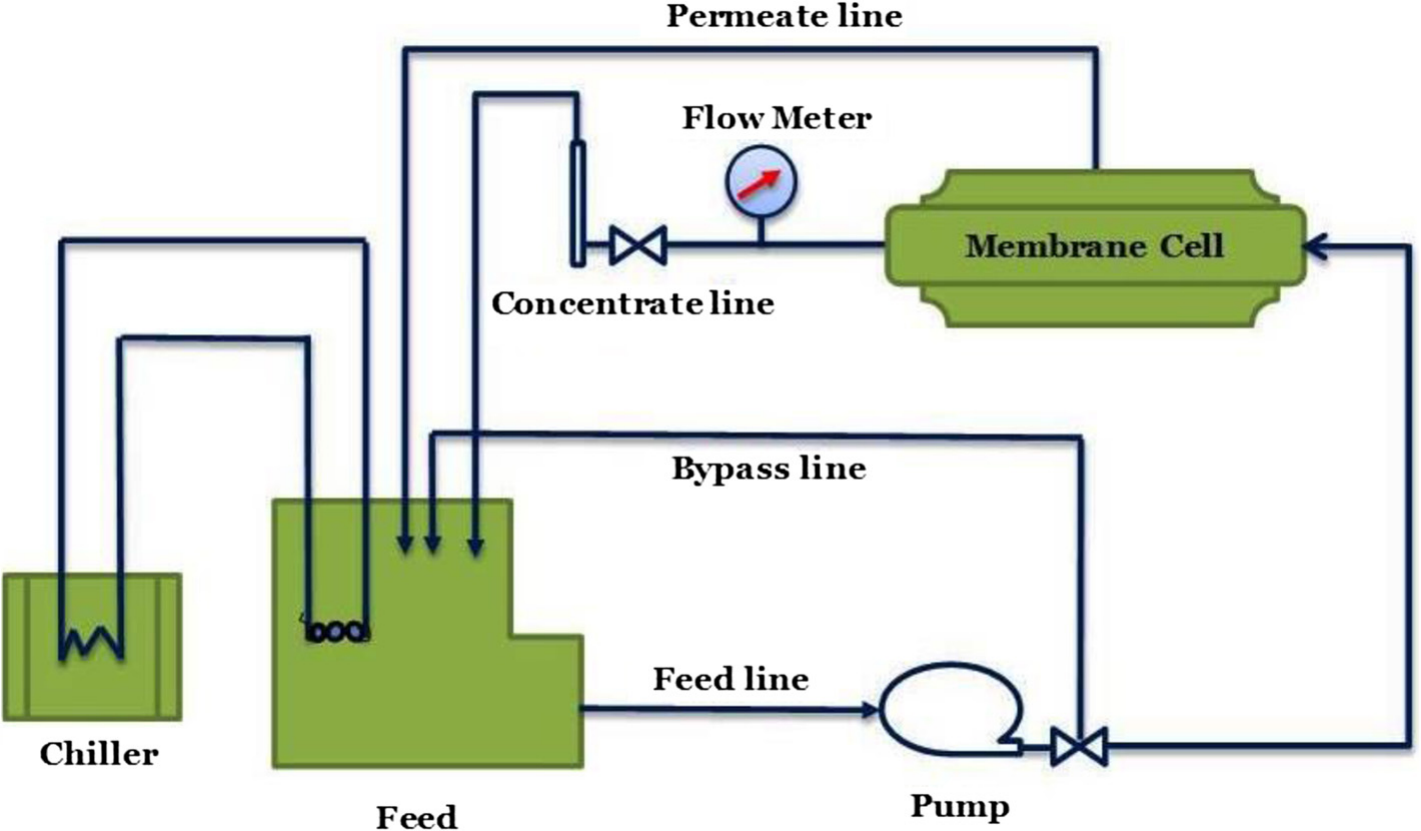
Schematic of the forward cross-flow filtration system

The performances of the prepared membranes were analyzed through a cross-flow system (CF042SS316 Cell, Sterlitech Corp., USA). The active membrane area in this system was 42 cm^2^. The feed water temperature was 25 °C with pH adjusted between 6 and 7, for a 2000 ppm NaCl concentration and a feed flow rate of 1 gallon per minute (gpm). The filtration was carried out at a transmembrane pressure of 225 psi. All measurements of the water flux and salt rejection were measured after 30 min of water filtration experiments to ensure steady-state operation had been reached. A schematic of the cross-flow filtration system is shown in Fig. 1.

The flux was calculated using the following equation [29].

1 $$ J=\left(\frac{1}{A}\right)\frac{d{V}_{\mathrm{p}}}{dt} $$

where *J* is the water flux (L/m^2^ h), *V*_p_ is the permeate volume (L), *A* is the membrane area (m^2^), and *t* is the treatment time (h). The salt rejection (*R*) was calculated using the following equation:

2 $$ R=\left(1-\frac{C_{\mathrm{p}}}{C_{\mathrm{f}}}\right)*100 $$

where *C*_p_ and *C*_f_ are the salt concentrations in the permeate and feed streams, respectively.

## Results and Discussion

The synthesized silver oxide nanoparticles were characterized by SEM, transmission electron microscopy (TEM), energy-dispersive spectroscopy (EDX), and X-ray diffraction (XRD) as follows.

**Fig. 2:**
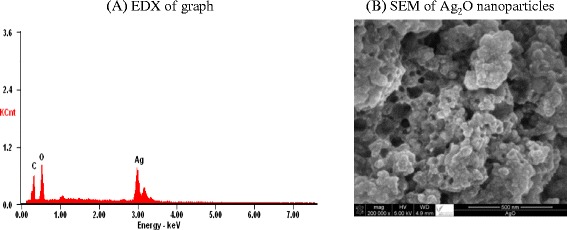
**a** EDX and **b** SEM of Ag_2_O nanoparticles

The size and surface morphology of Ag_2_O nanoparticles were determined by SEM, as shown in Fig. 2b, and the size of silver nanoparticles was estimated, in the form of nanocrystallites. EDX carried out during the SEM analysis conformed to the characteristic peaks of Ag, as shown in Fig. 2a.

**Fig. 3:**
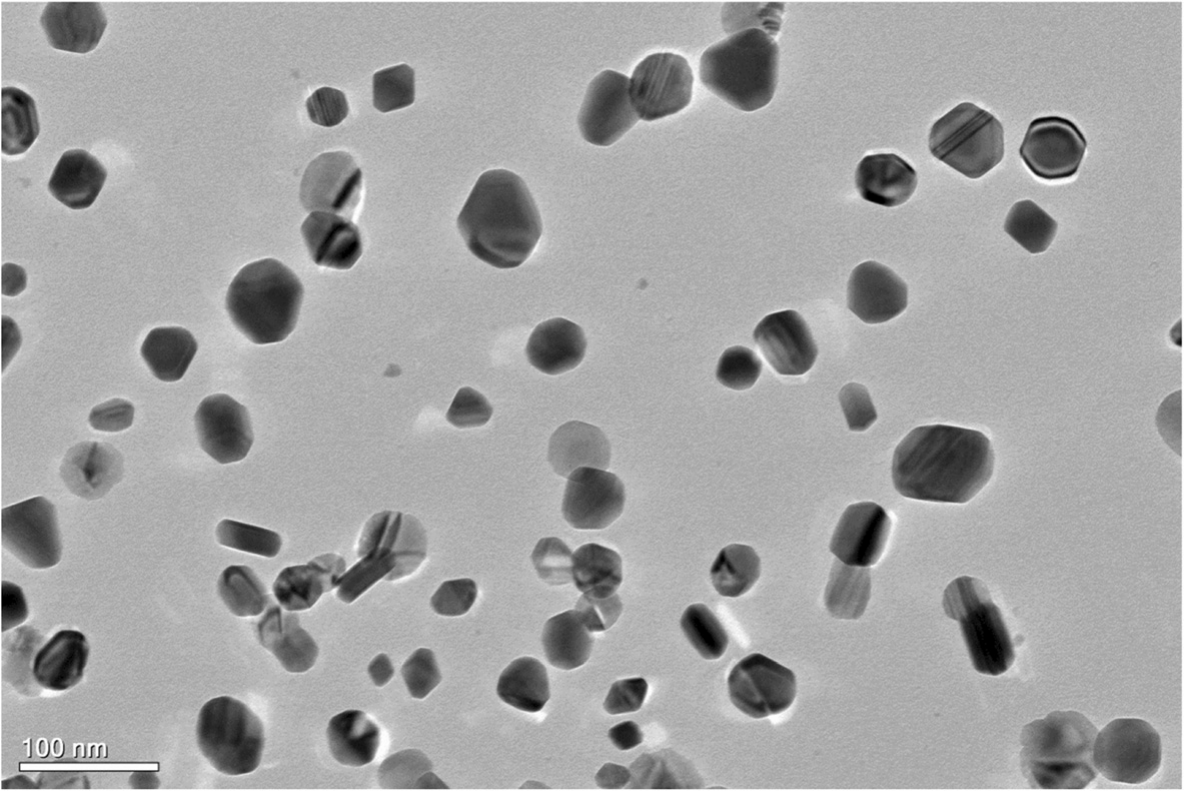
TEM of Ag_2_O nanoparticles

TEM analysis of Ag_2_O nanoparticles was also carried out to estimate the size of silver nanoparticles. Particle size was estimated to be in the range of 18 to 50 nm, confirming the results already estimated by SEM and EDX. TEM images are shown in Fig. 3.

**Fig. 4:**
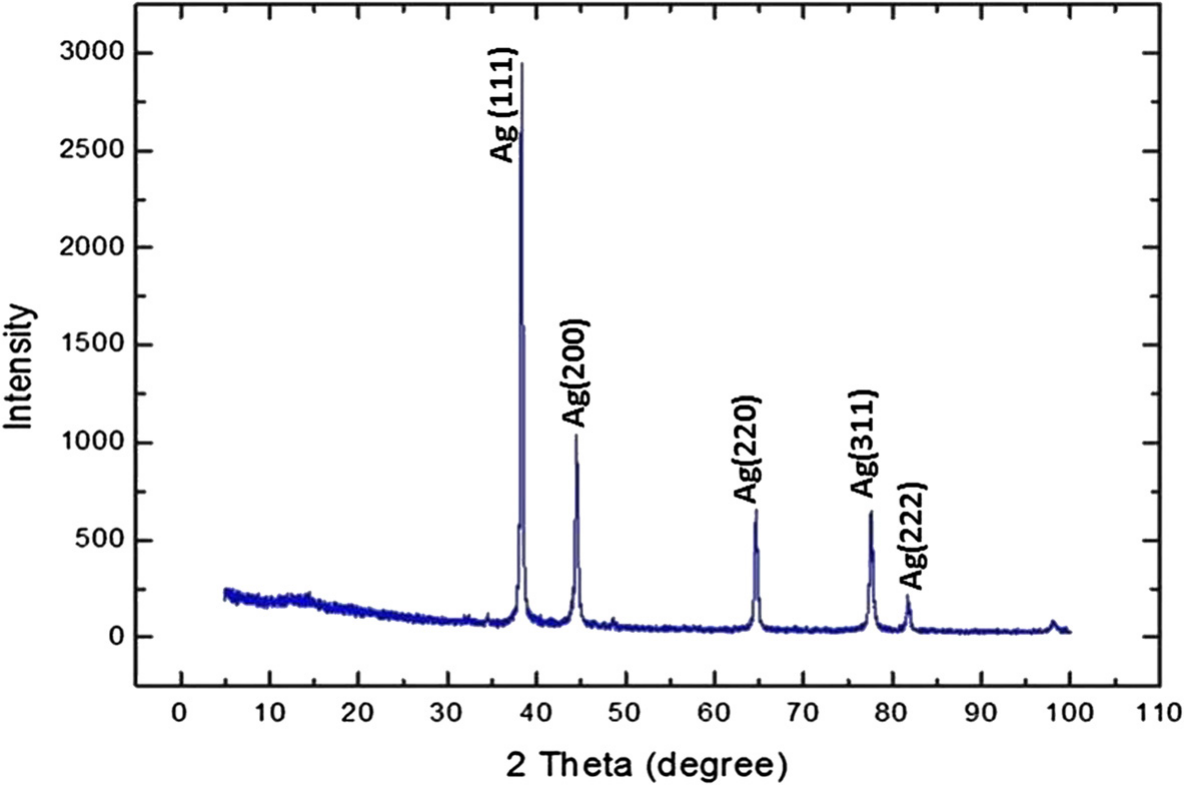
XRD patterns of Ag_2_O nanoparticles

Typical XRD spectra of the silver oxide nanoparticle powder carried out after thermal treatment are shown in Fig. 4. Five pronounced diffraction peaks were observed and that resemble the characteristics of Ag_2_O nanoparticles. Those five pronounced diffractogram peaks were found at 37.40° (111), 44.10° (200), 63.90° (220), 75.90° (311), and 80.90° (222). The average grain size was calculated using the Debye-Scherrer equation (*G* = 0.9*λ*/*β* cos*θ*), where *λ* is the X-ray wavelength (Cu, 1. 5418 Å), *θ* is the maximum of the Bragg diffraction peak, and *β* is the full width at half maximum. After a correction for instrumental broadening, the average value obtained for the crystallites was 45 nm [30].

**Fig. 5:**
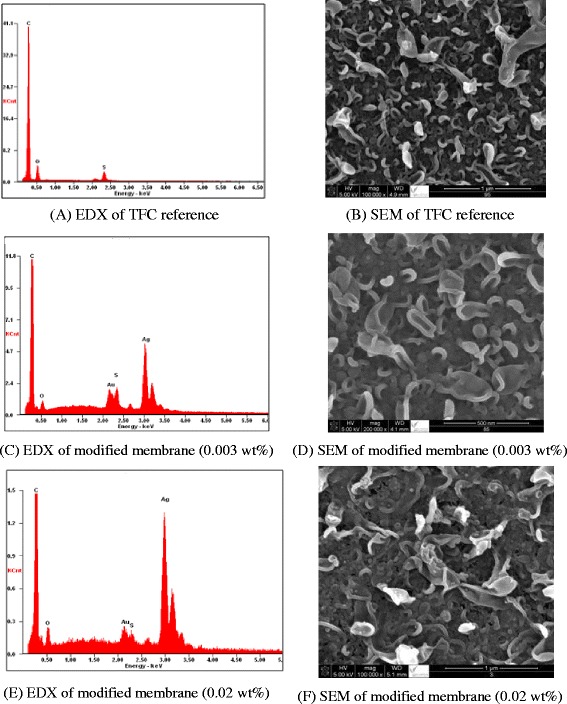
SEM images and EDX of TFC reference membranes and membranes modified with Ag_2_O nanoparticles. EDX (**a**) and SEM (**b**) of TFC reference. EDX (**c**) and SEM (**d**) of modified membrane (0.003 wt%). EDX (**e**) and SEM (**f**) of modified membrane (0.02 wt%)

The TFC membrane layer was found to be coated onto the PS support base during the interfacial polymerization (IP) reaction process between MPD and TMC, leading to the formation of a leaf-like morphology. SEM and EDX analysis was used to determine such morphology as well as the size of silver nanoparticles in the membranes. EDX spectra and SEM images of the reference TFC membrane are shown in Fig. 5a, b, respectively. Similarly, EDX spectra and SEM images of polymer membranes containing silver nanoparticles are shown in Fig. 5c, d, respectively. It was observed that impregnation of Ag_2_O nanoparticles in the polymer matrix does not affect the overall morphology of TFC membranes containing silver nanoparticles, even when the wt% range of Ag_2_O nanoparticles was increased to 0.02 wt% (Fig. 5f). However, partial aggregation of Ag_2_O nanoparticles was observed in the polymer matrix. The difference between the images clearly indicates the presence of Ag_2_O nanoparticles. This was further confirmed by EDX quantitative analysis, which clearly shows the presence of carbon, oxygen, and silver peaks as component elements. In comparison, a TFC reference membrane exhibited an ascendant and broadened ridge-valley structure, suggesting variation in surface roughness of TFC membranes under different nano-Ag_2_O loadings.

**Fig. 6:**
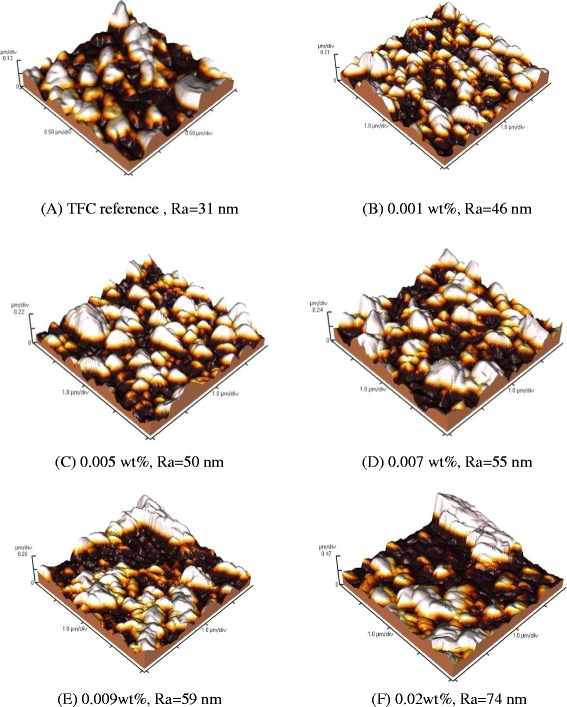
AFM images of membranes modified with silver oxide nanoparticles. **a** TFC reference, Ra = 31 nm. **b** 0.001 wt%, Ra = 46 nm. **c** 0.005 wt%, Ra = 50 nm. **d** 0.007 wt%, Ra = 55 nm. **e** 0.009 wt%, Ra = 59 nm. **f** 0.02 wt%, Ra = 74 nm

AFM analysis was carried out to further determine the morphology and roughness of the membrane surface after incorporation of silver oxide nanoparticles into the polymer membranes. AFM measurements of membranes containing different contents of silver oxide nanoparticles and are shown in Fig. 6. It can be observed that the roughness (*R*_a_) values increased with increasing silver oxide content in the polymer membranes. TFC membranes with 0.02 wt% silver showed the highest surface roughness (i.e., *R*_a_ = 74 nm), compared with that of a TFC reference membrane without silver content (*R*_a_ = 31 nm). This difference is presumed to be caused by the leaf-like shape as well as by the aggregation of Ag_2_O nanoparticles present in the membrane surface. The surface comprised continuous ridge and valley structures, confirming previous results obtained by other researchers [16, 20–22].

**Fig. 7:**
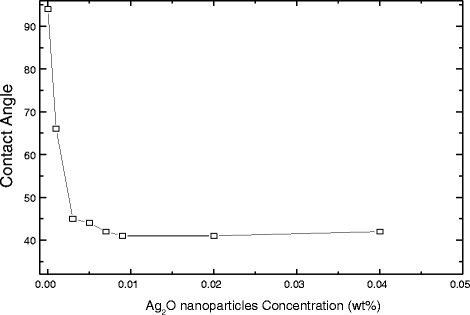
Contact angles of PA membranes containing different amounts of silver nanoparticles

Membranes containing different amounts of silver oxide nanoparticles were subjected to contact angle measurements to evaluate the hydrophilic and hydrophobic characteristics of the membranes; during contact angle measurements, reproducible standard deviation (SD) was taken into account with the ultimate aim of determining water permeate flux ability during the salt rejection process for underground water. An acceptable reproducible standard deviation was taken into account during the measurements. Contact angles, as measured with different PA membranes containing various amounts (wt%) of Ag_2_O nanoparticles, are presented in Fig. 7.

It can be observed that contact angle significantly decreased with increased silver oxide content in the membranes. For example, the presence of a silver content of 0.003 wt% in the membrane dropped the contact angle from 94° ± 1.8° to 45° ± 0.7°. Contact angles of the membranes did not change/decrease markedly above 0.01 wt% silver loading. Additionally, the hydrophilicity did not change significantly, despite the increasing content of Ag_2_O nanoparticles. Our results are in agreement with those obtained by other researchers [31]. It is known that there is a strong correlation between the geometry/stereochemistry of water at the solid-liquid interface and the hydrophilicity of the solid surface [32, 33]. Rearrangement of interfacial water molecules can increase hydrophilicity, thus improving water’s ability to form hydrogen bonds and, in turn, producing stronger interactions between water and the solid phase in the polymer matrix, i.e., the TFC surface.

The remarkable decrease in contact angle in the presence of embedded silver oxide nanoparticles can be explained as follows:

1. A large amount of embedded spherical Ag_2_O nanoparticles could have been exposed on the membrane surface. The hydrophilic nature of the silver nanoparticles would increase the hydrophilic character of the membrane surface and would also help water molecules to adhere to the Ag_2_O nanoparticles, through capillary effects [34]. This is consistent with the result of Jeong et al. [35], who observed that the contact angle of membrane surface decreased with increased zeolite content and attributed this to the super-hydrophilic property of the zeolite.
2. Second, silver oxide nanoparticles may hydrate and release heat when contacting with MPD aqueous solution [36]. This process may affect the IP reaction between MPD and TMC and, subsequently, may change the matrix of the PS support. If a large number of acyl chloride groups of TMC remained on the membrane surface without reacting with the amine groups of MPD, the hydrolysis of acyl chloride could generate carboxylic acid functional groups, which may lead to increased hydrophilicity [37]. It is known that the hydrophilic nature of Ag_2_O nanoparticles can significantly improve the overall hydrophilicity of membranes, thereby creating favorable conditions for improved water flux and antifouling. It is well understood that the morphological structure and the hydrophilicity of the membrane are the two main factors that govern the filtration properties of membranes and those of TFC membranes [38].

**Fig. 8:**
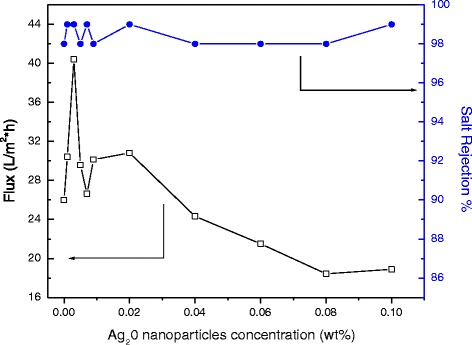
Water permeate fluxes and salt rejections of modified membranes containing various amounts of Ag_2_O nanoparticles

Polymer membranes containing various amounts of Ag_2_O nanoparticles were used to carry out a filtration process at 25 °C, using the cross-flow method. The results of water permeate fluxes and salt rejections for all membranes are presented in Fig. 8.

The maximum of the hybrid membranes’ water fluxes is greater than that of the TFC membranes (25 L/m^2^ h ± 2.3), where ±2.3 is the standard deviation of three measurements which was used to estimate the uncertainty of these measurements. The quantity of Ag_2_O nanoparticles was increased, and then the membrane water flux correspondingly increased up to a peak value at 0.003 wt% (40.43 L/m^2^ h ± 3.0) and then started to decrease. The peak value of water flux displays an enhancement of 160 % over that of the TFC reference membrane. On the other side, the salt rejection of both thin-film composite (TFC) and thin-film nanocomposite (TFN) membranes was established to be 98 % ± 0.7 and 99 % ± 0.1, respectively. The SD was measured for three measurements in each case. Consequently, the standard deviation of salt rejection for TFN was within 2 %. It is postulated that the improved water permeability may be ascribed to the nanometer pores in the silver oxide nanoparticles. On the other hand, the silver oxide nanoparticles may interrupt the interfacial polymerization method to amend the active layer permeability. Furthermore, the increased membrane surface roughness might relatively explain such flux enrichment. On the other hand, a high silver oxide loading may help to develop a comparatively thicker rejection polyamide layer, which decreased the water permeability and improved salt rejection. The transport properties of the membranes prepared in this study are listed in Table 1.

**Table 1:** Summary of membrane characteristics of present study

MPD	TMC	Additives	Conc. %	Salt rejection %	Flux (L/m^2^ h)
Constant at 2 %	Constant at 0.1 %	Ag_2_O	0.001	99 ± 0.1	30.43 ± 2.1
			0.003	99 ± 0.1	40.43 ± 3.0
			0.005	98 ± 0.3	29.57 ± 1.9
			0.007	99 ± 0.1	26.64 ± 2.2
			0.009	98 ± 0.2	30.14 ± 2.1
			0.02	99 ± 0.1	30.83 ± 2.9
			0.04	94 ± 0.7	24.33 ± 1.7
			0.06	98 ± 0.2	21.50 ± 2.2
			0.08	97 ± 0.9	15.43 ± 2.9
			0.10	99 ± 0.1	18.90 ± 2.1

*n*-Hexane (99 %), pressure (225 psi), and rate (1 gpm). The experimental data were reported as the average value of at least three repeated measurements in tests, error bars based on measurements of three coupons

Both permeate flux and salt rejection ability depend on the polyamide layer density, which is related to cross-linking density [39]. The polymer density across the barrier layer is not uniform [40]. The core layer (near the original MPD/TMC interface) is the most dense region, and the polymer density decreases gradually as the polymer grows further into the organic phase [41, 42]. In many applications of interfacial polymerization using MPD and TMC, the initial amine concentration is much higher than the acyl chloride concentration. Whether the amine concentration is decreased or the acyl chloride concentration is increased, this results in a more dense polyamide layer, compared with those prepared using higher amine/acid chloride molar ratios [43]. An increase in density or in thickness of the MPD/TMC barrier layer would increase the mass transfer resistance of the resulting membrane, thereby reducing permeate flux. Thus, varying the initial concentration of monomers can influence the membrane’s water and salt transport properties.

**Fig. 9:**
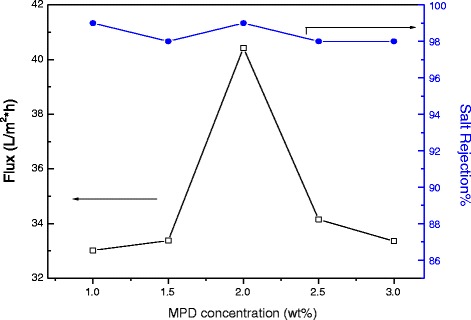
Water permeate flux and salt rejection of modified membranes containing fixed amounts of Ag_2_O nanoparticles at 0.003 wt% and TMC at 0.1 *w*/*v*%

**Fig. 10:**
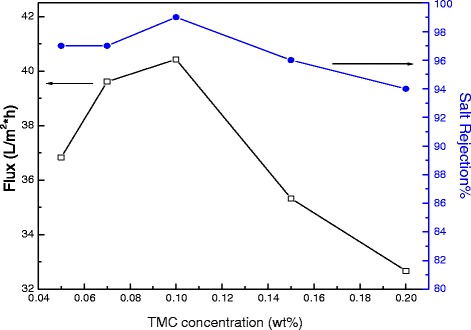
Water permeate flux and salt rejection of modified membranes containing fixed amounts of Ag_2_O nanoparticles at 0.003 wt% and MPD at 2 *w*/*v*%

TFC-modified membranes were prepared by varying the concentrations of MPD and TMC and keeping the mass of added Ag_2_O nanoparticles at 0.003 wt%. Figure 9 shows the influence of varying the MPD concentration on permeate flux and salt passage. Similarly, Fig. 10 presents the influence of changing the TMC concentration on flux and salt rejection.

Salt passage was found to be relatively insensitive to changes in MPD concentration. This suggests the formation of defect-free membranes in all cases. Maximum permeate flux was exhibited near 2 *w*/*v*% MPD. As the MPD concentration was increased, the driving force for MPD diffusion into the organic phase increased. Increased MPD concentration could, therefore, increase the barrier layer thickness and, thus, causes a lower permeate flux. As MPD concentration decreased, layer thickness was expected to decrease, which would tend to increase flux, but the resulting layer was also expected to become more dense as the molar ratio of amine/acyl chloride approached unity, which would lower flux [41, 44].

Figure 10 illustrates the influence of TMC concentration in the organic phase on permeate flux and salt passage at a constant MPD concentration of 2 *w*/*v*% in the aqueous phase, which was observed in Fig. 9 to be near the optimum flux value. Interfacial polymerization is typically MPD diffusion-controlled during growth of the polyamide layer. If TMC concentration is increased, the amine/acyl chloride molar ratio will decrease. This will, in turn, increase film density, causing a lower permeate flux [41, 44]. However, a decrease in permeate flux was also observed at the lowest TMC concentration of <0.1 %. Thus, the interfacial polymerization reaction is also reported to be TMC diffusion-limited [45]. A low concentration of acyl chloride groups in the reaction zone may allow the polyamide film to grow thicker, which would decrease flux [39].

**Fig. 11:**
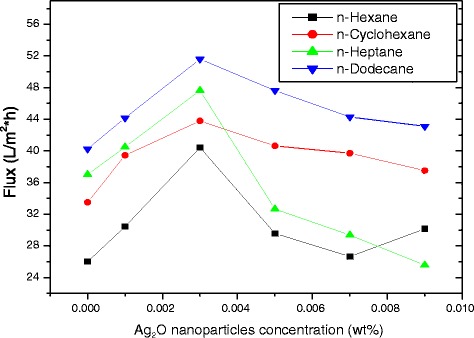
Water flux with different solvents

**Fig. 12:**
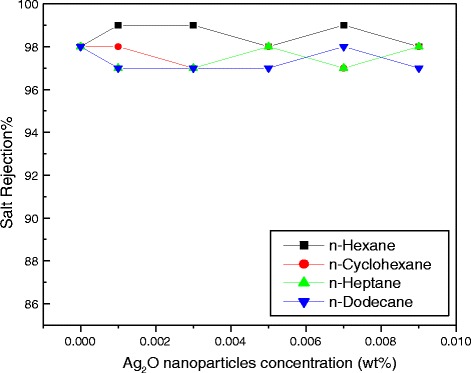
Salt rejection with different solvents

All the above experiments were carried out in a hexane medium. To investigate the influence of solvents on permeate flux and salt rejection, hexane was replaced with *n*-cyclohexane, *n*-heptane, and *n*-dodecane solvents. Figures 11 and 12 show the results for flux and salt rejection, respectively, and are in the order of *n*-dodecane > *n*-heptane > *n*-cyclohexane > *n*-hexane. However, the change in salt rejection with all these solvents was found to be within 2 % (Fig. 12).

There are many literature reports regarding the performance of interfacial polymerized commercial polyamide TFC membranes and of aromatic polyamide and TFC desalination membranes based on MPD and TMC [46–54]. The transport properties of the membranes prepared in this study were found comparable to those of the membranes listed in Table 2.

**Table 2:** Summary of membrane characteristics from previous studies

Nanoparticle type	Method	Modified membrane properties	Salt rejection	Flux (L/m^2^ h)	Ref.
MgTiO_3_ nanoparticles	IP (MPD/TMC)	Enhanced surface hydrophilicity, increase roughness	98	45	[46]
	MgTiO_3_ in TMC/hexane				
Zeolite nanoparticles	IP (MPD/TMC)	Smoother, hydrophilic, and negatively charged surfaces	93	40.2	[47]
	NaA in TMC/hexane				
Silica nanoparticles	IP (MPD/TMC)	Silica particle interacting well with the polyamide	91	27.2	[48]
	Silica in MPD/water				
Silica nanoparticles	IP (MPD/TMC)	Tunable pore radius and higher thermal stability	71.7	40.8	[49]
	Silica in MPD/water				
Silver nanoparticles	IP (MPD/BTC)	Higher anti-biofouling effect	97	94	[50]
	Silver in BTC				
TiO_2_ nanoparticles	The neat TFC membrane was dipped in the transparent TiO_2_ colloidal solution	Higher photocatalytic bactericidal efficiency under UV light	96.6	24.5	[51]
TiO_2_ nanoparticles	IP (MPD/TMC)	Enhanced surface hydrophilicity	95	9.1	[52]
	TiO_2_ in TMC and HCFC				
Zeolite nanoparticles	IP (MPD/TMC)	Increase roughness and contact angle	90.5	43.7	[53]
	NaY in TMC/hexane				
Al_2_O_3_ nanoparticles	IP (MPD/TMC)	Enhanced surface hydrophilicity	88	5	[54]
	Al_2_O_3_ in TMC/hexane				

## Conclusions

Silver oxide nanoparticles were embedded into the polyamide membrane through an interfacial polymerization process between MPD and TMC. EDX and SEM confirmed the formation of polyamide membranes embedded with silver oxide nanoparticles. EDX quantitative analysis confirmed the presence of silver oxide in polyamide components. Water flux and salt rejection performances revealed that the nanocomposite membrane was superior to the native (unmodified) membrane. Moreover, permeate flux was improved (from 26 to 40.5 L/m^2^ h), while salt rejection performances remained within 2 %.
